# Two Novel Lipophilic Antioxidants Derivatized from Curcumin

**DOI:** 10.3390/antiox11040796

**Published:** 2022-04-18

**Authors:** Tao Liu, Xiaohan Liu, Tosin M. Olajide, Jia Xu, Xinchu Weng

**Affiliations:** 1 School of Environmental and Chemical Engineering, Shanghai University, 381 Nanchen Road, Shanghai 200444, China; tao6218@shu.edu.cn (T.L.); tosmics@shu.edu.cn (T.M.O.); xuj_smile@163.com (J.X.); 2 School of Life Sciences, Shanghai University, 381 Nanchen Road, Shanghai 200444, China; 18800571978@163.com

**Keywords:** curcumin derivatives, antioxidant for high temperature, lipophilic, steric synergy effect

## Abstract

*Tert*-butyl curcumin (TBC), demethylated *tert*-butylated curcumin (1E,6E-1,7-bis(3-*tert*-butyl-4,5-dihydroxyphenyl)hepta-1,6-diene-3,5-dione, DMTC), demethylated curcumin (DMC), and Cur were synthesized from the starting compound, 2-methoxy-4-methylphenol. TBC and DMTC are two novel lipophilic compounds, and Cur and DMC are polar and hydrophilic. The antioxidant activities of Cur, TBC, DMC, and DMTC were evaluated by using the methods of 2,2-diphenyl-1-(2,4,6-trinitro-phenyl)-hydrazinyl (DPPH), deep-frying, and Rancimat. *Tert*-butylhydroquinone (TBHQ) and Butylated hydroxytoluene (BHT) were used as comparison compounds. Both Rancimat and deep-frying tests demonstrated that DMTC was the strongest antioxidant, and TBC also had stronger antioxidant activity than Cur. In the DPPH assay, DMC showed the highest scavenging activity, followed by DMTC, TBHQ, Cur, and TBC. DMTC and TBC can be potentially used as strong antioxidants in food industry, especially for frying, baking, and other high temperature food processing. DMTC is the strongest antioxidant in oil to our knowledge.

## 1. Introduction

Oxidation of lipids involves the generation of free radicals and induces food deterioration [1]. Usually, three common methods are used to prevent lipid oxidation: air isolation, temperature lowering, and addition of antioxidants during processing, transport, and storage [2]. However, addition of antioxidants is the most convenient, economical, and operable method in oil and food industry [3]. Free radicals, peroxides, and decomposition products produced by autoxidation are related to a variety of human diseases, such as cancers, cataracts, cardiovascular and cerebrovascular diseases [4,5,6]. Antioxidants are a class of substances that can retard deterioration caused by autoxidation of fats and oils, and fatty foods effectively at minute concentrations (< or = 0.02%) [7,8]. Although commercial synthetic antioxidants have been shown to have negligible toxicity at prescribed doses, natural antioxidants are widely considered safer and non-toxic [9,10]. The application of natural antioxidants is limited because most of their hydrophilic polyphenol structures make solubility in lipids difficult [11]. Over the years, a few researchers have modified the structures of natural antioxidants to improve their solubility in lipids [12].

Cur, a polyphenol, is isolated from the rhizome of turmeric (*Curcuma longa*) [13,14] and has numerous biological activities, such as anti-tumor, anti-oxidation, anti-inflammation, anti-HIV, and so on [15,16,17]. However, its application is limited in food industry because of poor fat solubility [18,19]. Therefore, using Cur as a lead compound to transform the structural properties of it has become a hot research topic, aiming to improve the structural disadvantages and expand the application scope. In recent years, some researchers have modified the structure of natural antioxidants to improve their solubility in lipids. Shi et al. [20] added *tert*-butyl to the *ortho*-position of the phenolic hydroxyl group of caffeic acid, which greatly improved the antioxidant activity of lipids. Olajide et al. [12] synthesized a lipophilic derivative of hydroxytyrosol with better steric synergy and stable structure, which enables it to meet the industrial requirements of food processing and bioactive ingredients under high temperature conditions. Some studies [21] have found that the hydrogen atoms on the adjacent α-CH_3_ can be used as functional hydrogen, because the hydrogen atoms on it can provide functional hydrogen to the adjacent phenolic hydroxyl group through intramolecular transfer, thereby improving the antioxidant activity of the compound. Our lab has analyzed the soybean-oil–water partition coefficient of TBHQ, caffeic acid, methyl caffeate, and butylated caffeic acid, and found that the introduction of tert-butyl group to the *ortho*-position of the phenolic hydroxyl group can indeed improve lipid solubility [22]. In order to improve the lipophilicity and antioxidant activity of Cur, two bulky apolar groups, *tert*-butyl groups were substituted at the *ortho*-position of the phenolic hydroxyl group (TBC), and the two methyl groups were further removed from TBC to become DMTC. Their antioxidant activities were evaluated by DPPH, deep frying, and Rancimat methods. There are few research reports on Cur as one of the antioxidants in food additives. The purpose of this study is to find an antioxidant with better fat solubility and strong antioxidant capacity in high-temperature-fried foods.

## 2. Materials and Methods

### 2.1. Chemicals and Reagents

2-methoxy-4-methylphenol, Vanillin, demethylvanillin, acetylacetone, tributyl borate, copper acetate, ethylene glycol, pyridine solution, boric anhydride, and *n*-butylamine, deuterium acetone, deuterium chloroform, 1,1-diphenyl-2-trinitrophenylhydrazine (DPPH) were purchased from Shanghai Macklin Biochemical Co., Ltd. (Shanghai, China). Phosphoric acid (86%), *tert*-butyl alcohol, anhydrous Na_2_SO_4_, AlCl_3_, hydrochloric acid (HCl), ethyl acetate (EtOAc), petroleum ether (PE), ethanol, acetone, chloroform, hydrogen peroxide, and methanol were all analytical grade and purchased from Sinopharm Shanghai Chemical Reagent Company.

Lard used in this experiment was rendered in the laboratory and stored at −18 °C. Commercial soybean oil and potatoes were purchased from Wilmar International Limited (Shanghai, China). Soybean oil was purified using column chromatography (PE as eluent) to remove antioxidants and other polar components, especially tocopherols and other phenolic compounds in the oil.

### 2.2. General Synthesis of Compounds

2-Methoxy-4-methyl-6-*tert*-butyl phenol: 2-methoxy-4-methylphenol (13.8 g; 0.1 mol) and 30 mL phosphoric acid (86%) were dissolved in 100 mL *tert*-butyl alcohol under stirring at 90 °C for 10 h and then deionized water was added to quench the reaction and the mixed solution was extracted with EtOAc, followed by washing with water (50 mL × 3). Then, the organic phase was collected, dried over anhydrous Na_2_SO_4_, and evaporated to obtain an oily mixture. The oily mixture was purified using column chromatography (PE/EtOAc, 20:1 *v*/*v*) and a pure compound (75% yield) was obtained as pale yellowish oily liquid. ^1^H NMR were (600 MHz, chloroform-d) δ1.39 (s, 9H), 2.28 (s, 3H), 3.84 (s, 3H), 5.82 (s, 1H), 6.58 (s, 1H), 6.69 (d, J = 0.35 Hz. 2H); ^13^C NMR (150 MHz, chloroform-d) δ 21.48; 29.48; 34.61; 56.12; 109.35; 119.37; 127.88; 135.17; 141.92; 146.46 (Figure S1).

*Tert*-butyl vanillin: 2-methoxy-4-methyl-6-*tert*-butyl phenol (4 g; 0.02 mol) and copper acetate (2 g; 0.01 mol) were dissolved in 80 mL ethylene glycol and heated to 100 °C for 36 h and then the reacted solution was extracted with EtOAc and further washed three times with water. Then, the organic phase was collected, dried over anhydrous Na_2_SO_4_, and evaporated in vacuum. The compound (70%) was obtained as light yellowish crystals after purification using flash chromatography (PE/EtOAc, 10:1 *v*/*v*). ^1^H NMR (600 MHz, chloroform-d) δ 1.44 (s, 9H), 3.97 (s, 3H), 6.59 (s, 1H), 7.33 (d, J = 1.65 Hz, 1H), 7.45 (d, J = 1.65 Hz, 2H), 9.82 (s, 1H). ^13^C NMR (150 MHz, chloroform-d) δ 29.15; 34.72; 56.37; 106.72; 125.37; 128.34; 135.65; 147.22; 150.35; 191.53 (Figure S2).

3-*Tert*-butyl-4,5-dihydroxybenzaldehyde: 2-methoxy-4-methyl-6-*tert*-butyl phenol (1.94 g, 0.01 mol) was dissolved in chloroform (60 mL), followed by the addition of AlCl_3_ (1.86 g, 0.014 mol) under nitrogen condition at 0 °C. After stirring for 5 min, pyridine solution (3.48 g) was slowly added. The mixture was refluxed at 80 °C for 24 h, and then 10% HCl (60 mL × 3) was added at room temperature. The organic phase was collected, dried over anhydrous Na_2_SO_4_, concentrated using rotovap, and the resulting product mixture was purified using column chromatography (PE/EtOAc/ methanol, 4:1:0.025 *v*/*v*) to afford compound (50% yield) as white acicular crystals. ^1^H NMR (600 MHz, chloroform-d) δ1.45 (s, 9H), 7.30 (d, J = 0.35 Hz.1H), 7.42 (s, 1H), 8.67 (s, 2H), 9.78 (s, 1H) (Figure S3).

**Scheme 1:**
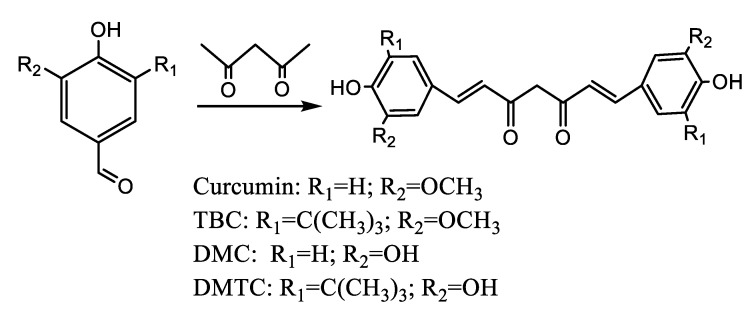
Synthetic route leading to Cur, TBC, DMC, and DMTC.

The compounds Cur, TBC, DMC, and DMTC were synthesized by condensation of vanillin, *Tert*-butyl vanillin, demethylvanillin and 3-*tert*-butyl-4,5-dihydroxy-benzaldehyde separately with acetylacetone based on the available methods [23] with some improvement. The condensation reaction was performed as expressed in Scheme 1: benzaldehyde (0.04 mol) was dissolved in anhydrous EtOAc (20 mL), heated to complete dissolution, cooled to 30 °C, and then tributyl borate (0.08 mol), boric anhydride (0.15 mol), and acetylacetone (0.02 mol) were added to the mixture. After stirring for 5 min, 1 mL *n*-butylamine (4 mL in total) was slowly added to the mixed solution system every 10 min. Then, the mixture was stirred for 4 h and left to stand overnight at 20 °C in the dark. In total, 30 mL of 0.4 N hydrochloric acid solution (60 °C) was added and stirred for 60 min. The mixed solution was extracted with EtOAc and then washed three times with water. The organic phase was collected, dried over anhydrous Na_2_SO_4_, and evaporated to obtain the reacted mixture, which was separated using column chromatography and the individual crude products were recrystallized from methanol at 4 °C.

Cur: 53% yield from vanillin using column chromatography (PE/EtOAc, 4:1 *v*/*v*), ^1^H NMR (600 MHz, acetone-d6) δ3.94 (s, 6H), 5.99 (s, 1H), 6.73 (s, 2H), 6.91 (s, 2H), 7.20 (d, J = 0.35 Hz.2H), 7.35 (s, 2H), 7.63 (s, 2H), 8.18 (s, 2H). ^13^C NMR (150 MHz, acetone-d6) δ5.43, 100.76, 110.66, 115.27, 115.35, 121.43, 122.95, 127.30, 140.52, 147.90, 183.65 (Figure S4).

TBC: 42% yield from *tert*-butylvanillin using column chromatography (PE/EtOAc, 8:1 *v*/*v*), ^1^H NMR (600 MHz, acetone-d6) δ 1.42 (s, 18H), 3.94 (s, 6H), 5.84 (s, 1H), 6.28 (s, 1H), 6.46 (s, 1H), 6.48 (s, 1H), 6.98 (s, 1H), 7.13 (s, 1H), 7.58 (s, 1H), 7.62(d, J = 0.35 Hz. 1H). ^13^C NMR (150 MHz, acetone-d6) δ 56.23, 100.85, 107.08, 121.19, 121.29, 126.06, 135.84, 141.27, 146.63, 146.97, 183.32. (Figure S5) ESIMS, *m*/*z* 480 [M]^+^ (Figure S6).

DMC: 30% yield from 3,4-dihydroxybenzaldehyde using column chromatography (PE/EtOAc, 1:1 *v*/*v*), ^1^H NMR (600 MHz, acetone-d6) δ5.98 (s, 1H), 6.62 (s, 2H), 6.89 (s, 2H), 7.08 (s, 2H), 7.19 (s, 2H), 7.54 (d, J = 0.35 Hz., 2H), 8.34 (s, 4H) (Figure S7).

DMTC: 14% yield from 3-*tert*-butyl-4,5-dihydroxybenzaldehyde using column chromatography (PE/EtOAc, 3:1 *v*/*v*), ^1^H NMR (600 MHz, acetone-d6) δ1.45 (s, 18H), 5.78 (d, J = 0.35 Hz, 1H), 6.43 (s, 1H), 7.09 (s, 1H), 7.10 (s, 1H), 7.30 (s, 2H), 7.42 (s, 2H), 7.52 (s, 1H), 9.78 (s, 2H) (Figure S8).

### 2.3. DPPH Test

The DPPH radical scavenging activities of BHT, TBHQ, Cur, TBC, DMC, and DMTC were evaluated according to a previous method reported by Shi et al. [20] with some modification. The concentrations of compounds in the reaction mixtures in ethanol were 1, 2.5, 5, 10, 20, 40, 50, 80, 100, 200, and 500 mg/L. A total of 0.5 mL of varying concentrations of compounds was mixed with 2.5 mL ethanolic solution of DPPH (0.1 × 10^−3^ mol/L). The mixtures were shaken vigorously and stored in a dark chamber to react for 30 min, and then the reducing absorbance (Ai) of DPPH was read at 517 nm on a UV-2450 spectrophotometer (Shimadzu Corp, Kyoto, Japan). The radical scavenging activities, expressed as EC_50_, are the effective concentrations of the compounds required to obtain 50% antioxidant capacity. DPPH free radical scavenging activities of the compounds were calculated as below:

(1) $${\mathrm{EC}}_{50}\;\mathrm{or}\;\mathrm{Scavenging}\;\mathrm{activity}\;\left(\%\right)=\left(1-\frac{\mathrm{Ai}-\mathrm{Aj}}{A_{0}}\right)\times 100\%\;$$

where Ai represents the absorbance of the mixture of 0.5 mL varying concentrations of compound solution and 2.5 mL DPPH free radical ethanol solution; Aj represents the absorbance of the mixture of 0.5 mL ethanol solution and 2.5 mL DPPH free radical ethanol solution; A_0_ represents the absorbance of 0.5 mL of varying concentrations of compound mixed with 2.5 mL ethanol solution.

### 2.4. Rancimat Test

The antioxidant activities of BHT, TBHQ, Cur, TBC, DMC, and DMTC in lard were examined based on the previously described method by Shi et al. [20] on Rancimat 743 apparatus (Metrohm, Herisau, Switzerland). Three-gram lard samples containing 0.01% and 0.02% of the compounds mentioned above were, respectively, added into the Rancimat sample tubes. Rancimat test conditions of acceleration oxidation were: air flow rate fixed at 20 L/h and temperatures fixed at 100, 110, 120, 130, and 140 °C.

The results of Rancimat tests are expressed as induction period (IP) and protection factor (Pf), which was determined based on the method reported by Olajide et al. [12]:

(2) $$\mathrm{Pf}=\frac{{\mathrm{IP}}_{\mathrm{sample}}}{{\mathrm{IP}}_{\mathrm{control}}}\;$$

where IP_sample_ and IP_control_ represent the induction period of oxidation in lard containing antioxidant and without antioxidant, respectively. Pf < 1 means that the compound has pro-oxidant activity. Pf = 1 means the compound has neither antioxidant activity nor pro-oxidant activity [24]. The 1 < Pf < 3 indicates that the compound has antioxidant activity and Pf > 3 indicates that the compound has strong antioxidant activity. Pf > 6, the compound is defined here to have very strong antioxidant activity.

### 2.5. Deep Frying Test

The 0.02% compounds were added to 800 g of purified soybean oil, respectively. There were six experimental samples (BHT, TBHQ, Cur, TBC, DMC, and DMTC) and a control sample. All seven samples were heated to 180 ± 5 °C, then 30 ± 3 g potatoes were fried every hour for 10 min (10 h/day for 3 days) under atmospheric pressure. During the experiment, there was no new oil added. Oil samples (30 g) were taken every 3 h and stored in a −18 °C refrigerator. Finally, the conjugated dienes (CD) and acid values (AV) of the oil sample were determined following the IUPAC method [25].

CD: Oil sample (1 mg) was dissolved in *n*-hexane (100 mL), then the absorbance was measured at 233 nm using a V-1600PC UV-Vis spectrophotometer. The CD was calculated based on its absorbance value as below:

(3) CD =(A/C)× P

Among them, A represents the absorbance value, C represents the concentration of oil sample (g/100 mL), and P represents the thickness of the quartz glass dish used for measurement (1 cm).

AV: Oil sample (10 g), diethyl ether-isopropanol mixture solution (*v*/*v*: 1:1; 80 mL) and 3 drops of phenolphthalein indicator (1 g phenolphthalein dissolved in 95% ethanol solution (100 mL)) were added into the conical flask, then shaken thoroughly to dissolve. Then, 0.01 mol/L KOH standard titrated aqueous solution was used to manually titrate the sample solution until the sample solution was slightly red at first. When there was no obvious fading within 15 s, it was the end point of the titration. The titration was stopped immediately and the number of milliliters of standard titration solution consumed by the titration was recorded. The method for the control is as above without the oil sample. The AV was calculated as below:

(4) $$\mathrm{AV}\;\left(\mathrm{mg}/g\right)=\frac{\left(V-V_{0}\right)\times c\times 56.1}{m}$$

Among them, V represents the volume of the standard titration solution consumed by the sample measurement (mL); V_0_ represents volume of standard titration solution consumed by the corresponding control determination (mL); c represents molarity of standard titration solutions (0.01 mol/L); 56.1 represents molar mass of potassium hydroxide (g/mol); m represents oil sample weight (g).

### 2.6. Statistical Analysis

The statistical analysis was conducted using IBM SPSS 22.0 and Excel. All experiments were performed in duplicate, and values were expressed as mean ± standard deviation (SD). One-way analysis of variance (ANOVA) with a Tukey’s HSD posthoc test were applied to BHT, TBHQ, Cur, TBC, DMC, and DMTC evaluations to determine significant differences (*p* < 0.05) between samples.

## 3. Results

### 3.1. DPPH Test

Antioxidants can provide hydrogen atoms to the DPPH free radicals to become non-free radicals [26]. This is observed in real time as the violet DPPH alcoholic solution fades into light yellow or colorless. EC_50_ can represent the antioxidant strength of the compounds and the stronger the antioxidant activities, the lower the EC_50_ value.

TBC, DMC, and DMTC compared to BHT and TBHQ were determined and calculated (Table 1). The scavenging DPPH free radical ability of antioxidants mainly depends on their number of functional hydroxyl groups and speed to provide hydrogen atoms to DPPH free radicals. The EC_50_ of DMC (10.0) was the lowest and meant the highest scavenging activity because of the highest functional hydroxyl group weight per 100 g of compound. DMTC (12.3) exhibits higher than TBHQ (19.5) but weaker activity than DMC (10.0). BHT (81.0) is the least active one.

**Scheme 2:**
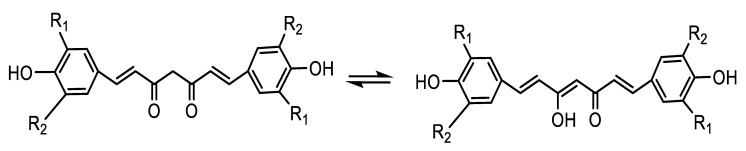
Elucidation of tautomerization structures of Cur and its derivatives.

The EC_50_ values of DMC (10.0) are higher than DMTC (12.3) which is in turn higher than TBHQ (19.5), whereas that of TBC (73.1) was much lower than Cur (40.5). TBC differs from Cur in that a *tert*-butyl substituent is inserted at the *o*-position of the phenolic hydroxyl group, which increased the steric hindered effect of the compound and delayed the release rate of hydrogen greatly, resulting in its slow rate of free radical capturing and also reduced functional hydroxyl group ratio of the compounds. Although phenolic hydroxyl group weight per 100 g of antioxidants (PHW/100 g A) of TBHQ (20.5) is very close to that of DMC (20.0), its diketone structure can easily tautomerize to enol structure. Here, all Cur and its derivatives have enolic hydroxyl groups, but BHT and TBHQ do not; therefore, Cur and its derivatives scavenge one more DPPH free radical as seen in Scheme 2 and Table 1. The FHW/100 g A (phenolic hydroxyl group + enolic hydroxyl group) of DMC (30.0) is much larger than that of TBHQ (20.5) although their PHW/100 g A values are very close. The scavenging DPPH free radical power decreased as follows: DMC (EC_50_ = 10.0, FHW/100 g A = 30.0) > DMTC (12.3, 22.5) > TBHQ (19.5, 20.5) > Cur (40.5, 13.8) > TBC (73.0,10.6) > BHT (81.0, 7.7). The order of scavenging DPPH free radical powers of the antioxidants agrees with that of their FHW/100 g A completely (Table 1).

### 3.2. Rancimat Test

The antioxidant effects of Cur, TBC, DMC, and DMTC were compared to BHT and TBHQ at various temperatures (100, 110, 120, 130, and 140 °C) and concentrations of 0.01 and 0.02% by the Rancimat method separately. The results are expressed as Pfs of samples relative to the stability of lard without antioxidants added [24].

At concentrations of 0.01 and 0.02%, the Pfs of Cur and its derivatives rose with the increase in concentrations. Statistical analysis of these results reveals a significant effect (*p* < 0.05) for Cur, TBC, DMC, and DMTC under different concentrations (0.01% and 0.02%). Pfs and IPs were determined to evaluate the antioxidant activities of the different compounds directly and accurately. All Pfs of the lard samples containing the compounds (Table 2) were larger than 1, indicating that all compounds have antioxidant activity, and higher values correspond to stronger antioxidant activity. Generally, the amounts of antioxidants added to food should be less than or equal to 0.02% in the edible oil industry according to the regulation. Pf of DMTC was 22.0 (>> 6) which shows that DMTC is a very strong antioxidant, and much higher than that of TBHQ. DMTC is the strongest antioxidant in lard, to our knowledge, when 0.02% of it was added.

**Scheme 3:**
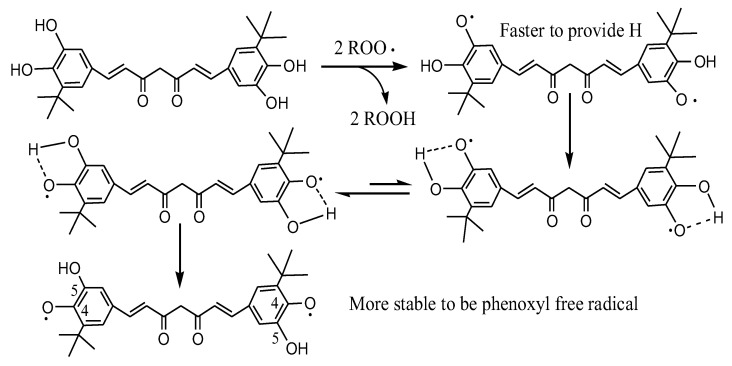
Elucidation of the synergistic effects of *ortho*-hydroxyl groups on DMTC.

DMTC is *tert*-butylated DMC, so its fat solubility is greatly improved. DMTC also has a perfect steric synergic effect so that the hydroxyl group at the 5-position can easily and quickly provide hydrogen to active free radicals. Then, the antioxidant free radical itself changes to more stable 4-phenoxy free radical by tautomerization as seen in Scheme 3 which explains the steric synergy very well. The antioxidant activities of compounds increased in the following order: Control < BHT < Cur < TBC < TBHQ << DMC << DMTC.

Table 3 shows that Pfs of 0.02% of BHT, TBHQ, Cur, TBC, DMC, and DMTC decreased as the heating temperature increased from 100 to 140 °C. One-way ANOVA followed by Tukey’s HSD poshoc test indicated significant differences (*p* < 0.05) for TBHQ, DMC, and DMTC under different temperatures (100, 110, 120, 130, and 140 °C). The Pfs of BHA, Cur, and TBC showed no significant difference (*p* > 0.05) by temperature. The Pfs of 0.02% of BHT and TBHQ, which are widely used lipid antioxidants commercially, decreased from 2.2 and 9.2 to 1.4 and 4.6, respectively, when the temperature increased from 100 to 140 °C. The Pfs of Cur, TBC, DMC, and DMTC decreased from 2.7, 3.4, 11.4, and 22.0 to 1.5, 1.9, 6.0, and 12.0, respectively. DMTC still has high Pfs (12.0) even at 140 °C, much higher than the others. The antioxidant capacities of each compound decreased when the temperature increased from 100 to 140 °C. However, the antioxidant activities of the compounds at same temperature were ranked in the following order: Control < BHT < Cur < TBC < TBHQ << DMC << DMTC. The antioxidant activity of DMTC is the strongest in oil to our knowledge.

### 3.3. Deep Frying Test

Fried foods are quickly and conveniently prepared by deep frying. People love fried foods due to their crispy texture and unique flavor. However, high temperature leads to oxidation and hydrolysis of oils, and other reactions, resulting in harmful substances for human health. Antioxidants can effectively slow down the oxidation rate of oils. In this study, the antioxidant activities of Cur, TBC, DMC, and DMTC were compared with TBHQ which is used very often in commercial frying oils.

**Figure 1:**
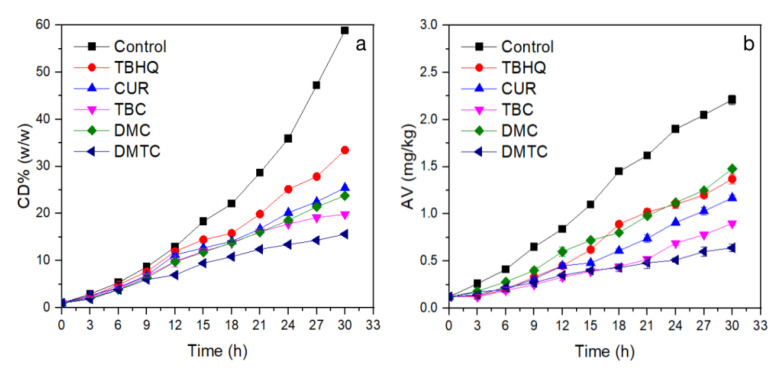
Changes in conjugated dienes (CD) and acid values (AV) of soybean oil spiked with or without antioxidants during deep frying at 180 °C. (**a**) CD, (**b**) AV. (n = 2).

Autoxidation of unsaturated fatty acids is a free radical chain reaction and poly double bonds will be conjugated to form conjugated dienes (CD) as oxidized [27]. When frying at high temperature (>180 °C), peroxides are decomposed quickly, and free fatty acids are also formed. Therefore, the peroxide value (PV) cannot indicate the stabilities of frying oil at high temperatures (>180 °C) well. The CD value and acid value (AV) are usually used to determine the degree of oxidation of frying oil under high temperature. As can be seen from Figure 1a, the content of CD increased continuously as the frying time lengthened. The CD value of the control group was clearly higher than those antioxidants added and indicated that the addition of antioxidants could effectively inhibit lipid oxidation. During the first 9 h of frying, the CD values of samples changed slowly, because the antioxidant played a vital role. After more than 12 h frying, however, differences in antioxidant activity began to appear as antioxidants were consumed. DMTC (CD = 15.6%) exhibited the best antioxidant activity when the frying time reached 30 h, and Cur and its derivatives were obviously better than TBHQ (33.5%) at high temperature due their higher molecular weight. TBHQ is unstable and volatile at high temperatures, which reduces its antioxidant activity during frying [28,29]. The antioxidant activities of DMC and DMTC in Rancimat tests were much higher than that of Cur and TBC, but in the high temperature frying experiment, DMC does not suppress CD generation efficiently and this result may be related to its low solubility in oil and more DMC was absorbed by potato chips which are mainly carbohydrates, moisture, and proteins. TBC has better solubility in oils than DMC, so shows better antioxidant activity.

Acid value (AV), as an important index to measure the quality of oil, reflects the content of free fatty acids in oil [30,31]. The AV is generally used as one of the quality parameters. Large AV means that the oil is hydrolyzed and/or oxidized significantly to release fatty acids. The standard of commercial edible oils limits the AV; therefore, smaller AV is required for edible oils. The main methods of increasing AV in oils contain: (1) unsaturated fatty acids undergo oxidation reactions to form hydroperoxides and further decomposed to aldehydes and some of them eventually oxidize to small free fatty acids; (2) fatty acids which are hydrolyzed from acylglycerides. According to Figure 1b, inhibition of free fatty acids produced by rancidity by different antioxidants is shown as follows: Control < TBHQ ≈ DMC < Cur < TBC < DMTC.

## 4. Discussion

In the present research, Cur always attracted wide attention due to its uniquely rare diketone colored symmetric molecular structure, containing one central methylene, one *β*-diketone, two unsaturated double bonds, and two phenolic hydroxyl groups [14]. With the improvement in Cur structure, there are more and more studies on the structure–activity relationship between Cur structure and antioxidant activity [32]. Studies have found that the phenolic hydroxyl group of Cur plays an important role in antioxidant activity, and the strength of antioxidant activity is related to the number of phenolic hydroxyl groups [33]. Moreover, the position of the phenolic hydroxyl group on the benzene ring also affects the antioxidant activity of Cur to a certain extent [34]. Rukkumain found that phenolic hydroxyl groups at the 2-position had higher antioxidant activity than hydroxyl groups at the 4-position, and catechol structure had higher antioxidant activity than hydroquinone [35]. Other studies have found that the introduction of a methyl group in the o-position of the phenolic hydroxyl group can also improve the antioxidant activity of Cur [36]. The results were consistent with those of Cur, TBC, DMC, and DMTC in the DPPH experiment, Rancimat and deep-frying tests. In the DPPH experiment, it was obvious that more phenolic hydroxyl groups in the unit molecular weight of the compound would lead to stronger DPPH radical scavenging, and DMC and DMTC with catechol structure had more obvious scavenging effects. DMTC is *tert*-butylated DMC, so its fat solubility is greatly improved. DMTC also has a perfect steric synergic effect where the hydroxyl group at 5-position can easily and quickly provide hydrogen to active free radicals and then the antioxidant free radical itself changes to a more stable 4-phenoxy free radical by tautomerization as seen in Scheme 3, which explains the steric synergy very well. That is the main reason why DMTC and BTC have stronger antioxidant activity than others in oils.

## 5. Conclusions

In this work, two novel lipophilic derivatives of Cur, TBC and DMTC, were synthesized and their antioxidant activity was evaluated using the methods of DPPH, Rancimat, and deep-frying. Results revealed that the antioxidant activities of the two novel Cur derivatives were better than Cur. DMTC showed the strongest antioxidant activity in oil due to the presence of more phenolic hydroxyls and a bulky *tert*-butyl group on the *o*-position of hydroxyl group, both of which play important roles when it acts as an antioxidant in oil. Moreover, DMTC is by far the strongest antioxidant in lard tested by the Rancimat method to date. TBC and DMTC could be potentially used as powerful antioxidants in food industry. Previously, we only studied the antioxidant activity of TBC and DMTC in oil, and their toxicity and effects on oil quality need further study.

## Figures and Tables

**Table 1:** The EC_50_ values and the weight of phenolic or functional hydroxyl groups per 100 g of the antioxidant compounds.

Compounds	EC_50_ (mg/L)	PHW/100 g A * (g/100 g)	FHW/100 g A ^#^ (g/100 g)
BHT	81.0 ± 0.37 ^a^	7.7	7.7
TBHQ	19.5 ± 0.30 ^d^	20.5	20.5
Cur	40.5 ± 0.18 ^c^	9.2	13.8
TBC	73.0 ± 0.09 ^b^	7.1	10.6
DMC	10.0 ± 0.01 ^e^	20.0	30.0
DMTC	12.3 ± 0.10 ^e^	15.0	22.5

PHW/100 g A *: phenolic hydroxyl group weight (g)/100 g antioxidant compounds; FHW/100 g A ^#^: functional hydroxyl group weight (g)/100 g antioxidant compounds. Values are expressed as mean ± standard deviation (S.D.). Statistical analyses included a one-way ANOVA with Tukey’s HSD posthoc test. Different superscript letters indicate that differences are significant at *p* < 0.05.

**Table 2:** Protection factors (Pf) of Cur and its derivatives at various concentrations (0.01 and 0.02%) at 100 °C.

Conc (%)	Pf					
	BHT	TBHQ	Cur	TBC	DMC	DMTC
0.01	1.82 ± 0.18 ^g^^h^	7.92 ± 0.45 ^d^	1.41 ± 0.05 ^h^	2.08 ± 0.11 ^gh^	6.87 ± 0.43 ^e^	13.36 ± 0.23 ^b^
0.02	2.22 ± 0.05 ^gh^	9.16 ± 0.32 ^d^	2.72 ± 0.02 ^fg^	3.36 ± 0.18 ^f^	11.35 ± 0.21 ^c^	22.04 ± 0.27 ^a^

A total of 3.00 g of lard was used; IP of lard: 4.28 ± 0.12. Values are expressed as mean ± standard deviation (S.D.). Statistical analyses included a one-way ANOVA with Tukey’s HSD posthoc test. Different superscript letters indicate that differences are significant at *p* < 0.05.

**Table 3:** Protection factors (Pfs) of Cur and its derivatives (0.02%), and comparison of antioxidants at various temperatures (100, 110, 120, 130, and 140 °C).

Tem (°C)	BHT	TBHQ	Cur	TBC	DMC	DMTC
100	2.2 ± 0.05 ^op^	9.2 ± 0.32 ^gh^	2.7 ± 0.02 ^op^	3.4 ± 0.18 ^no^	11.4 ± 0.21 ^ef^	22.0 ± 0.27 ^a^
110	2.0 ± 0.07 ^op^	7.8 ± 0.21 ^hij^	2.3 ± 0.10 ^op^	2.8 ± 0.12 ^op^	9.9 ± 0.14 ^fg^	19.0 ± 0.79 ^b^
120	1.8 ± 0.09 ^p^	6.4 ± 0.20 ^jkl^	1.9 ± 0.13 ^op^	2.2 ± 0.10 ^op^	8.2 ± 0.02 ^hi^	15.6 ± 0.52 ^c^
130	1.6 ± 0.18 ^p^	5.4 ± 0.14 ^lm^	1.7 ± 0.21 ^p^	1.9 ± 0.06 ^op^	7.1 ± 0.21 ^ijk^	13.4 ± 0.33 ^d^
140	1.4 ± 0.46 ^p^	4.6 ± 0.49 ^mn^	1.5 ± 0.21 ^p^	1.9 ± 0.14 ^op^	6.0 ± 0.26 ^klm^	12.0 ± 0.29 ^de^

Values are expressed as mean ± standard deviation (S.D.). Statistical analysis included a one-way ANOVA with Tukey’s HSD posthoc test. Different superscript letters indicate differences are significant at *p* < 0.05.

## Data Availability

Data is contained within the article.

## Supplementary Materials

The following are available online at https://www.mdpi.com/article/10.3390/antiox11040796/s1, Figure S1. 1H and 13C NMR spectrum of 2-Methoxy-4-methyl-6-tert-butyl phenol; Figure S2. 1H and 13C NMR spectrum of tert-butyl vanillin; Figure S3. 1H and 13C NMR spectrum of 3-tert-butyl-4,5-dihydroxybenzaldehyde; Figure S4. 1H and 13C NMR spectrum of curcumin; Figure S5. 1H and 13C NMR spectrum of tert-butyl curcumin; Figure S6. The high resolution mass spectrometry of tert-butyl curcumin; Figure S7. 1H and 13C NMR spectrum of demethylated curcumin; Figure S8. 1H and 13C NMR spectrum of 1E,6E-1,7-bis(3-tert-butyl-4,5- dihydroxyphenyl)hepta-1,6- diene-3,5-dione.
