# Supplementary material for: Two Novel Lipophilic Antioxidants Derivatized from Curcumin
Source: Antioxidants (Basel). 2022 Apr 18;11(4):796. doi: 10.3390/antiox11040796 (PMC9033154; doi:10.3390/antiox11040796)
Supplement: Supplementary file 1 [file antioxidants-11-00796-s001.zip › antioxidants-1648099-supplementary.pdf]

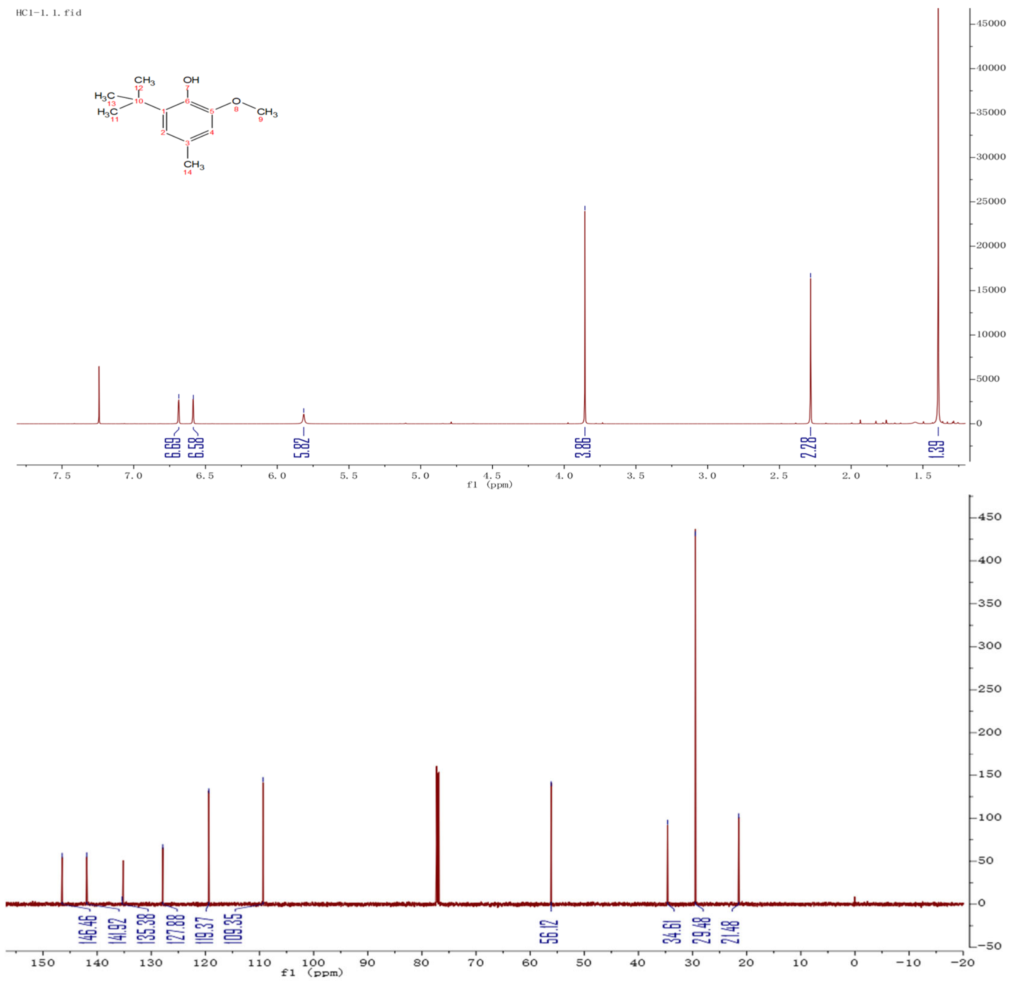

Figure S1. ^{1}H and ^{13}C NMR spectrum of 2-Methoxy-4-methyl-6-*tert*-butyl phenol

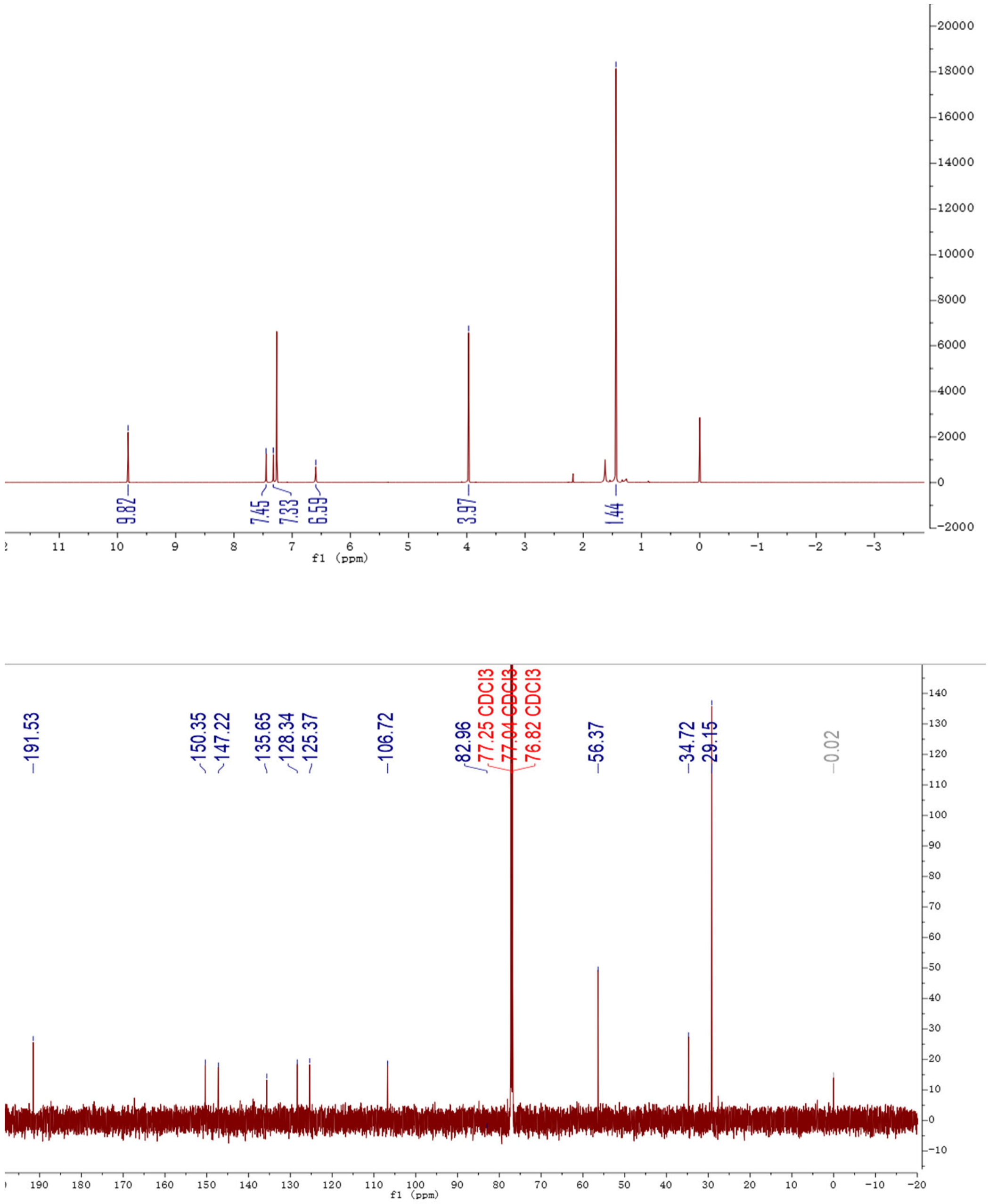

Figure S2. ^{1}H and ^{13}C NMR spectrum of *tert*-butyl vanillin

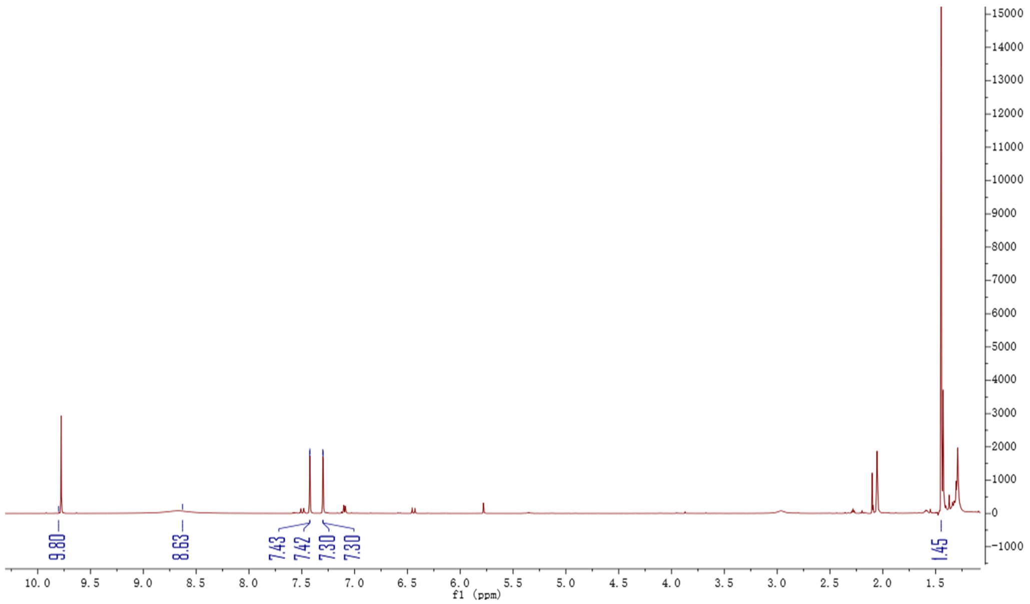

Figure S3. ^{1}H and ^{13}C NMR spectrum of 3-t*ert*-butyl-4,5-dihydroxybenzaldehyde

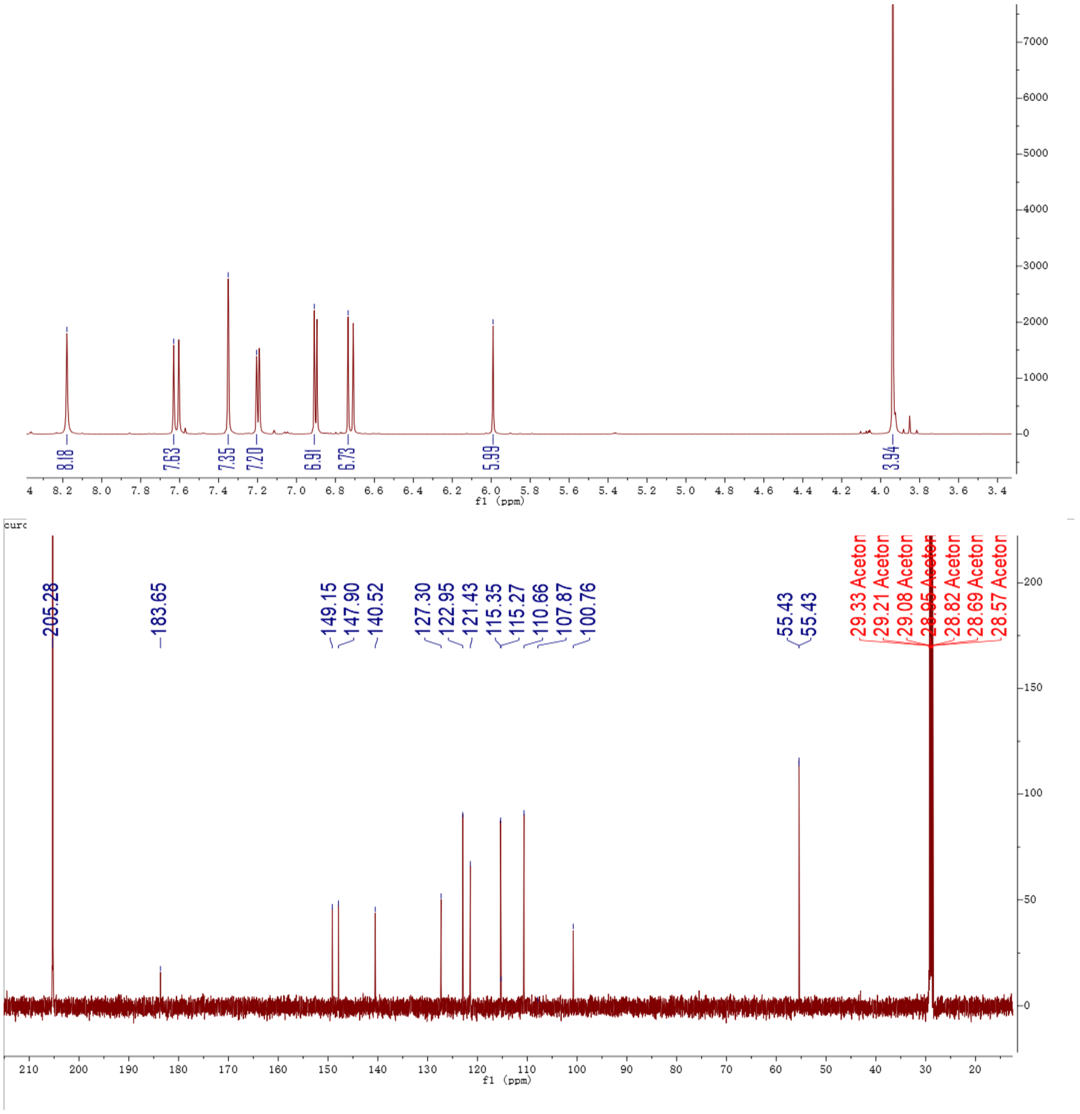

Figure S4. ^{1}H and ^{13}C NMR spectrum of curcumin

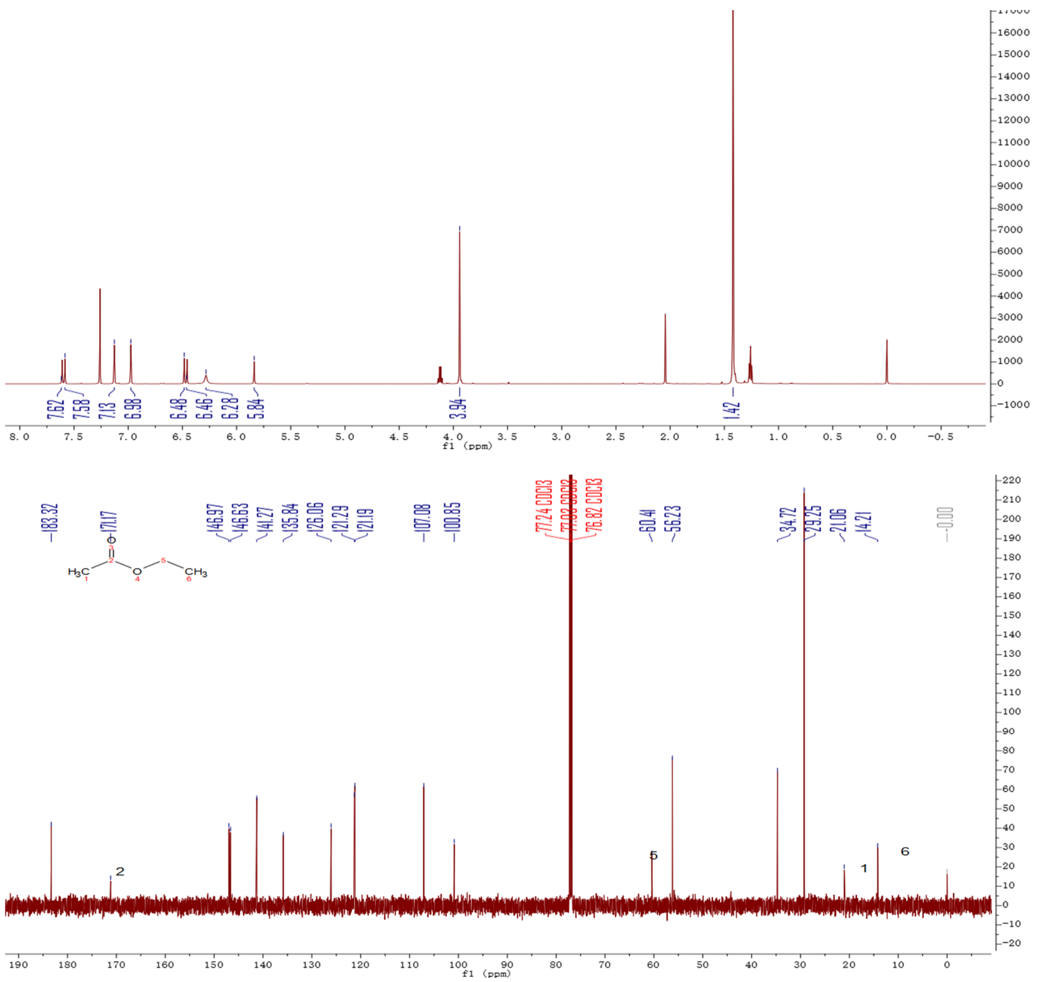

Figure S5. ^{1}H and ^{13}C NMR spectrum of *tert-butyl* curcumin

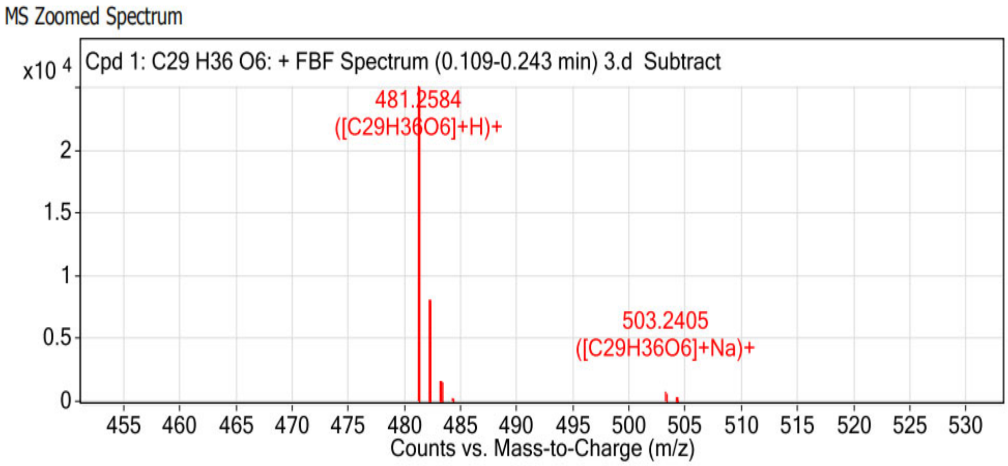

Figure S6. The high resolution mass spectrometry of *tert-butyl* curcumin

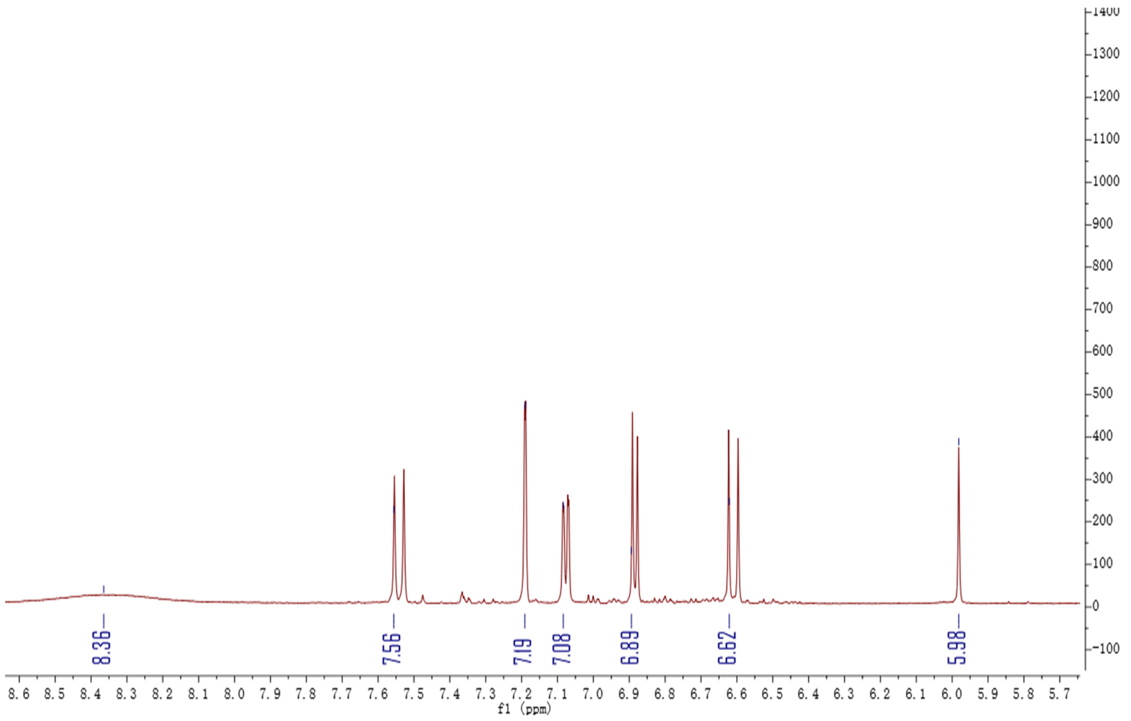

Figure S7. ^{1}H and ^{13}C NMR spectrum of demethylated curcumin

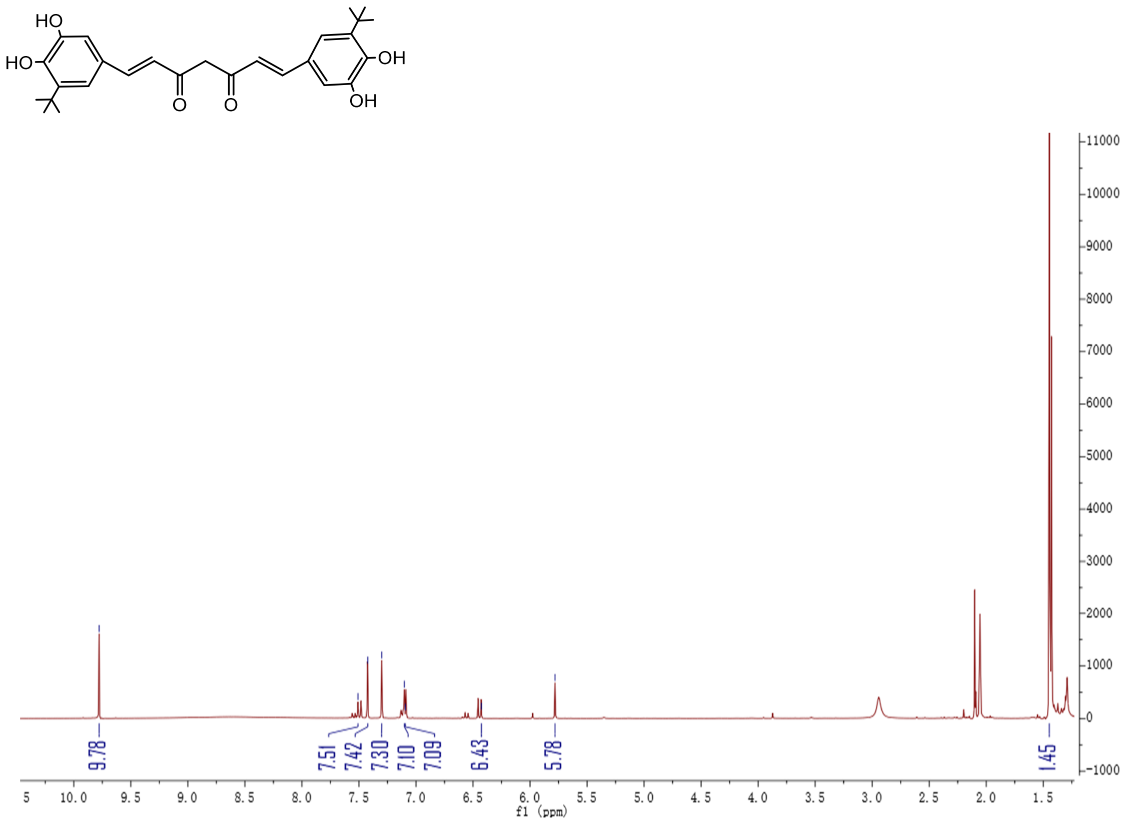

Figure S8. ^{1}H and ^{13}C NMR spectrum of 1E,6E-1,7-bis(3-*tert*-butyl-4,5-dihydroxyphenyl)hepta-1,6- diene-3,5-dione
